# Increased Fidelity Reduces Poliovirus Fitness and Virulence under Selective Pressure in Mice

**DOI:** 10.1371/journal.ppat.0010011

**Published:** 2005-10-07

**Authors:** Julie K. Pfeiffer, Karla Kirkegaard

**Affiliations:** Department of Microbiology and Immunology, Stanford University School of Medicine, Stanford, California, United States of America; Scripps Research Institute, United States of America

## Abstract

RNA viruses have high error rates, and the resulting quasispecies may aid survival of the virus population in the presence of selective pressure. Therefore, it has been theorized that RNA viruses require high error rates for survival, and that a virus with high fidelity would be less able to cope in complex environments. We previously isolated and characterized poliovirus with a mutation in the viral polymerase, 3D-G64S, which confers resistance to mutagenic nucleotide analogs via increased fidelity. The 3D-G64S virus was less pathogenic than wild-type virus in poliovirus-receptor transgenic mice, even though only slight growth defects were observed in tissue culture. To determine whether the high-fidelity phenotype of the 3D-G64S virus could decrease its fitness under a defined selective pressure, we compared growth of the 3D-G64S virus and 3D wild-type virus in the context of a revertible attenuating point mutation, 2C-F28S. Even with a 10-fold input advantage, the 3D-G64S virus was unable to compete with 3D wild-type virus in the context of the revertible attenuating mutation; however, in the context of a non-revertible version of the 2C-F28S attenuating mutation, 3D-G64S virus matched the replication of 3D wild-type virus. Therefore, the 3D-G64S high-fidelity phenotype reduced viral fitness under a defined selective pressure, making it likely that the reduced spread in murine tissue could be caused by the increased fidelity of the viral polymerase.

## Synopsis

RNA viruses have the highest error rates in nature, resulting in the likelihood that each virus differs from other viruses in the population by one or more mutations. The consequence of this “infidelity” is that the viral population as a whole, under selective pressure from the immune system or antiviral drugs, may benefit from adaptive changes in a subset of its members. Therefore, it has been theorized that RNA viruses need high error rates to survive in complex environments. We tested this hypothesis using a drug-resistant poliovirus that contains a mutation in its polymerase that reduces errors during replication. We found that this high-fidelity mutant virus has reduced growth in mice, a complex environment where mutations may be required for growth and spread within the infected animal. At least part of this attenuation is likely due to the high fidelity of this mutant virus, since it was unable to compete with the low-fidelity version of the virus in the context of a defined selective pressure. Therefore, it is likely that mutations do benefit viral populations, especially in complex environments such as an infected animal or human.

## Introduction

RNA viruses have the highest replicative error rates in nature, with approximately one mistake made per 1,000–100,000 nucleotides copied [1–3]. As a result, each viral genome is likely to differ from every other virus by one or more point mutations. The complex populations thus generated, called quasispecies, have been hypothesized to be important for survival of the population as a whole in the presence of selective pressure, under which a few viruses that contain beneficial mutations would survive and act as founders for the next generation [1,4]. For example, viruses with mutations that confer resistance to neutralizing antibodies would benefit the virus population as a whole under selective pressure from the host immune response. However, the vast majority of errors made during replication are deleterious, resulting in debilitation of a high percentage of the population. Therefore, RNA viruses live on the edge of “error catastrophe,” where the cost of deleterious mutations is in dynamic balance with the benefit of adaptive mutations [1,5,6].

A promising new class of antiviral drugs, RNA virus mutagens, takes advantage of the cost of error catastrophe. Ribavirin is a nucleoside analog mutagen, which is incorporated into the viral genome during replication and increases error frequency by promiscuous base-pairing [3]. Ribavirin debilitates the viral population over several rounds of replication by causing accumulation of deleterious mutations and error catastrophe [5]. Previously, we isolated a poliovirus variant able to survive serial passage in ribavirin, and mapped this phenotype to a single mutation in the viral polymerase, 3D-G64S [7]. The mechanism of ribavirin resistance in 3D-G64S mutant poliovirus was shown to be due to increased fidelity of RNA replication by showing that it has lower reversion frequency at the resistance mutation for the anti-viral compound guanidine [7], confers resistance to other mutagens as well [7], by direct sequencing of viral genomes [8], and by demonstration that the purified 3D-G64S poliovirus displays increased fidelity in solution that correlates in a delayed conformational change before phosphoryl transfer compared to the wild-type enzyme [8].

If a viral quasispecies is important to the survival either of individual genomes or of the population during growth under standard laboratory conditions, the higher fidelity of 3D-G64S virus should result in an attenuated phenotype. Therefore, we wondered if the 3D-G64S virus would have growth defects in tissue culture, a relatively simple environment, or in mice, a complex environment with many selective pressures. Laboratory strains of mice are not susceptible to poliovirus infection; however, mice transgenic for the human poliovirus receptor, CD155, are susceptible to injected poliovirus [9–11]. When poliovirus receptor (PVR) mice are given poliovirus by intramuscular (IM) injection in the leg, animals develop paralysis and eventually die of paralytic disease. Poliovirus inoculated by IM injection is thought to traffic up the sciatic nerve, to the spinal cord, and then to the brain [12,13], during which many different environments are likely to play a role in its propagation.

In this study, we found that 3D-G64S poliovirus is less pathogenic than wild-type virus in mice, but displays little growth defect in tissue culture. This attenuation in mice was at least partially due to the increased fidelity of the 3D-G64S virus in the context of a defined selected pressure, since it was unable to compete with 3D wild-type virus in the context of a revertible attenuating point mutation. Therefore, we argue that, in the face of the myriad selective pressures encountered by poliovirus during murine infection, the decreased variability in the population generated by the relatively high-fidelity 3D-G64S polymerase may be a distinct disadvantage for the growth and spread of the mutant virus.

## Results

### 3D-G64S Virus Is Less Pathogenic than Wild-Type Virus in Mice

To test whether the pathogenicity of high-fidelity 3D-G64S virus was comparable to that of wild-type virus in infected animals, mice that express the human poliovirus receptor [9] were inoculated by IM injection with 5 × 10^6^ PFU (plaque-forming units), an amount slightly over the LD50 for these animals [9]. Animals infected in this way develop symptoms similar to poliomyelitis in humans [10]. First, animals display lethargy, then weakness and paralysis in the inoculated leg, then paralysis of both legs, followed quickly by death. For this study, we monitored the time required for the first symptoms, weakness and paralysis of the inoculated limb, to develop. Figure 1 shows time courses for 48 mice inoculated with either wild-type or 3D-G64S virus. The 3D-G64S-inoculated animals developed disease significantly more slowly than wild-type infected animals, and fewer animals succumbed to disease (Figure 1). Therefore, the 3D-G64S virus is less pathogenic than wild-type virus in mice.

**Figure 1:**
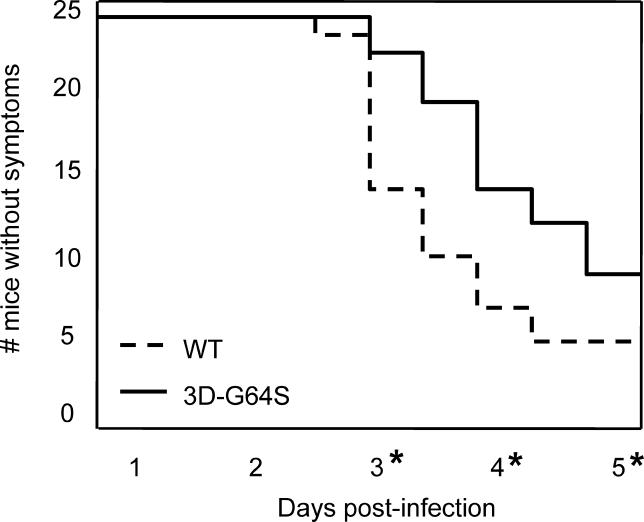
Pathogenesis of Wild-Type and 3D-G64S Viruses in PVR Mice PVR mice were given IM injections with 5 × 10^6^ PFU of either the wild-type or 3D-G64S viruses and were monitored for symptoms of disease. Mice were scored as having symptoms when weakness was first observed in the inoculated limb. Asterisks denote time points where statistically significant differences (*p* < 0.05, determined by two-sample Student's *t-*test [d 3, 0.0025; d 4, 0.0053; d 5, 0.043]) were observed between 3D-G64S and wild-type viruses. DOI: 10.1371/journal.ppat.0010011.g001

### Competition between 3D-G64S and Wild-Type Virus in Mice

To test whether the decreased pathogenicity of the 3D-G64S virus could be observed in mixed infections, we infected PVR mice with mixtures of wild-type and 3D-G64S virus. A 1:2 ratio of wild-type:3D-G64S virus was chosen because, as will be shown in subsequent figures, this ratio was required to achieve equal RNA yields in mixed infections of HeLa cells. A total of 2 × 10^7^ total PFU of mixed virus were given as IM injections and mice were sacrificed at the first sign of disease, usually 3 or 4 d post-infection. To monitor the relative accumulation of the mutant and wild-type RNAs of mixed infections, we took advantage of the presence of an additional BstBI restriction enzyme site engineered at the position of the 3D-G64S mutation. As shown in Figure 2A, BstBI digestion of end-labeled RT-PCR reaction products from mixtures of wild-type and 3D-G64S RNAs is expected to produce products of different sizes. For the sets of primers used here, a 558-basepair (bp) fragment is produced from RT-PCR analysis of wild-type RNA, and a 396-bp product from 3D-G64S RNA. Although this assay was not designed to quantify the total amounts of these viral RNAs, it should accurately determine their relative proportions within a population. Inoculated leg muscles and brains were harvested from mice infected with a mixture of wild-type and 3D-G64S viruses, RNA was extracted, and the products of reverse transcription, PCR amplification, end-labeling and BstBI digestion were quantified. As shown in Figure 2B and quantified in Figure 2E, both wild-type and 3D-G64S viruses were able to replicate within the inoculated muscle tissue. However, when products from brains were analyzed (Figures 2C and 2F), a distribution of outcomes was observed: in many mice (69%), only the wild-type band was present (e.g., mice 1, 2, 4, 6); in some mice (22%), the 3D-G64S band was predominately or exclusively present (e.g., mouse 5), and only rarely (9%) were both bands present (e.g., mouse 7). These results support two conclusions; first, 3D-G64S virus has a defect in spreading to the brain following IM infection, and second, because usually only one of the two viruses was found in the brain, there is a bottleneck between the injection site and the brain. This bottleneck to poliovirus spread has been observed previously in both humans [14,15] and in mice [15] (J. K. Pfeiffer, unpublished data).

**Figure 2:**
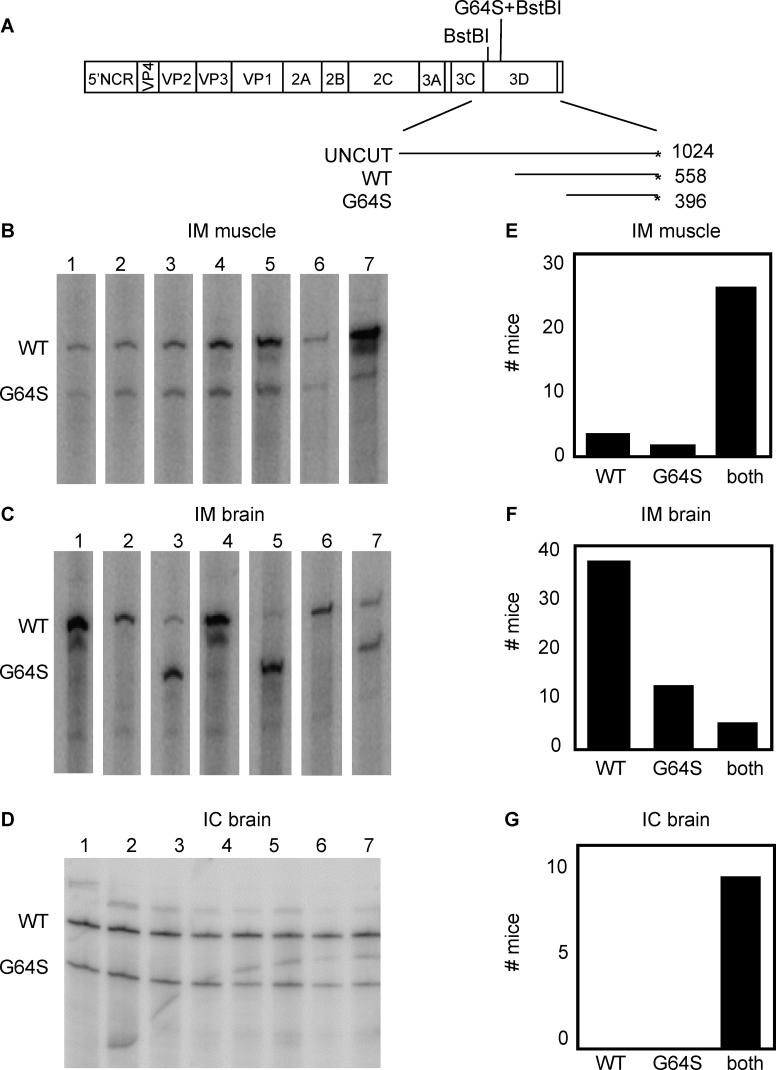
Viral Competition Assay in Poliovirus Receptor-Expressing Mice (A) Diagram of poliovirus genome showing BstBI restriction enzyme sites within the coding region for 3D polymerase. Digestion of 1,024-bp ^32^P end-labeled (*) RT-PCR products yields a 558-bp labeled band for wild-type and a 396-bp labeled band for 3D-G64S. 6-wk-old mice were given IM injections with a 1/2 mixture of wild-type to 3D-G64S poliovirus (2 × 10^7^ PFU total virus per mouse). Tissues were harvested when animals first developed paralysis, between days 3 and 5. PCR products were end-labeled with ^32^P during synthesis by inclusion of a radiolabeled downstream primer and products were digested with BstBI before electrophoresis on a denaturing polyacrylamide gel. Products from muscle samples from the IM infections are shown in (B) while products from brain samples of the IM infections are shown in (C). In (D), 2-wk-old mice were given IC injections with a 1/1 mixture of wild-type to 3D-G64S virus, and brain tissue was harvested when mice became lethargic or paralyzed. For (B), (C), and (D), representative gels are shown. In (E), (F), and (G) all mouse data obtained from the types of experiments shown in (B), (C), and (D), respectively, are quantified, and show the total number of mice whose tissues contained predominately wild-type virus, 3D-G64S virus, or both. The total number of mice per condition are as follows: IM-muscle samples, 31 mice; IM-brain samples, 54 mice; IC-brain samples, 10 mice. DOI: 10.1371/journal.ppat.0010011.g002

To ensure that both viruses were able to replicate and be detected in the brain at the same time, intracerebral (IC) inoculations were performed using a 1:1 input ratio of wild-type:3D-G64S virus. Samples from the brains of these mice (Figures 2D and 2G) showed that both viruses were able to replicate in the brain when inoculated into this tissue. Therefore, the preferential spread of wild-type virus from the infected muscle to the brain did not result from an inability of 3D-G64S virus to replicate in murine brain tissue.

### 3D-G64S Displays a Slight Growth Defect in Cultured Cells

The decreased virulence of the high-fidelity 3D-G64S mutant virus (Figure 1) could result from: (1) an advantage conferred to the wild-type virus by the increased complexity of its quasispecies population, or (2) a growth disadvantage conferred to the 3D-G64S mutant virus by some other property of the mutant polymerase than its increased fidelity. To monitor the replication of high-fidelity 3D-G64S poliovirus in tissue culture, several kinds of viral growth assays were performed. After infection of HeLa cell monolayers with wild-type and 3D-G64S virus at a multiplicity of infection (MOI) of 10 PFU/cell, the growth curves of single-cycle infections were found to be indistinguishable (Figure 3A). Furthermore, when wild-type and 3D-G64S virus growth was compared in plaque assays (Figure 3B), the plaques were of similar size at all temperatures tested, indicating equivalent growth of the viruses for several rounds of replication in HeLa cells.

**Figure 3:**
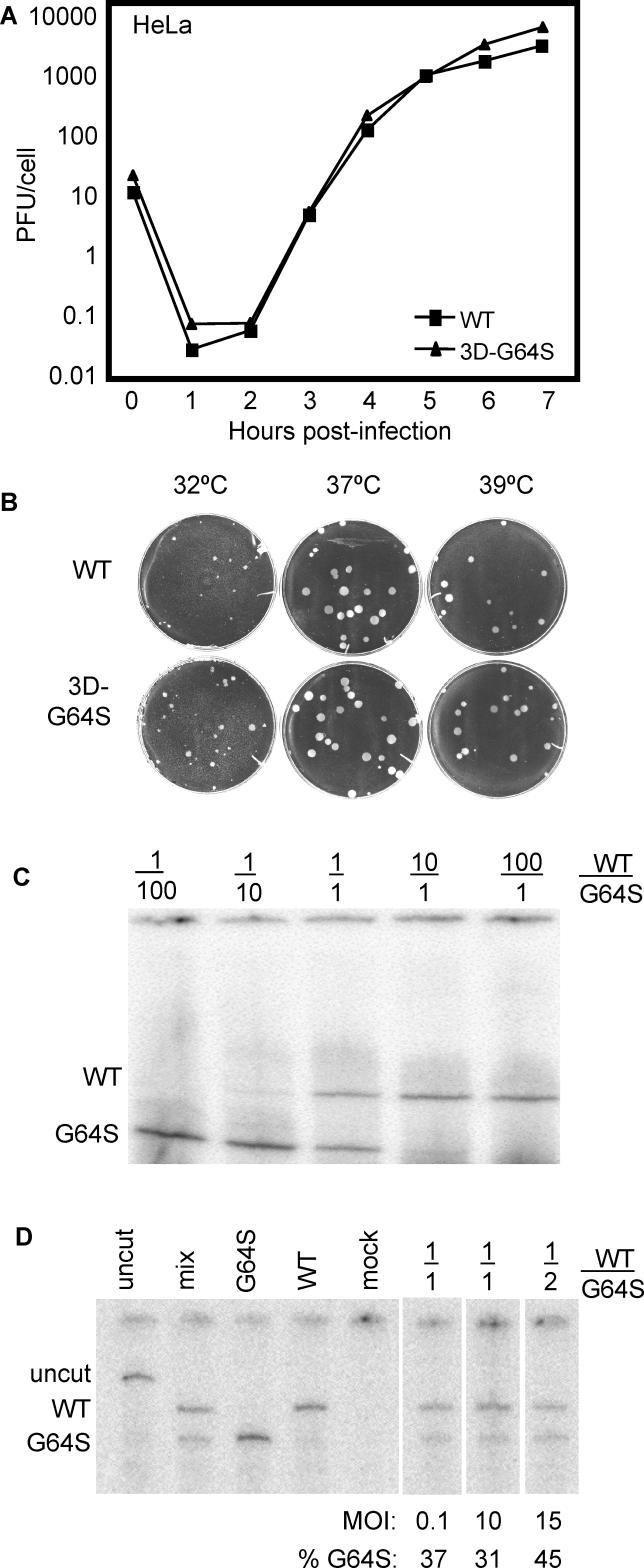
Growth of Wild-Type and 3D-G64S Poliovirus in HeLa Cells (A) Single-cycle growth curve. 2 × 10^6^ HeLa cells were infected at an MOI of 10 PFU/cell with either wild-type or 3D-G64S viruses at 37 °C. Cell-associated virus was harvested at indicated times and titered by plaque assay. (B) Plaque assays. Wild-type or 3D-G64S poliovirus was plated on HeLa cell monolayers and incubated under an agar overlay for 48 h at indicated temperatures before staining the cells with crystal violet. (C) Competition assay titration with plasmid DNA. Plasmid DNAs that encode wild-type and 3D-G64S poliovirus were diluted and mixed at the ratios indicated: for example, 1/100 denotes a ratio of 0.1 ng of wild-type viral cDNA to 10 ng of 3D-G64S cDNA. End-labeled PCR products were digested with BstBI and were electrophoresed on denaturing polyacrylamide gels. (D) Competition assay in HeLa cells infected with wild-type and 3D-G64S virus. HeLa cells (2 × 10^6^) were infected at an MOI of 0.1 or 10 PFU/cell, as indicated, with wild-type virus, 3D-G64S virus, or both, and cells were harvested 5 h after infection. RNA was extracted, subjected to RT-PCR as in (C), and digested with BstBI. Control bands “uncut” and “mix” were amplified from cDNAs. Products from MOI 0.1 single infections with wild-type, 3D-G64S- and mock-infected cells are shown, as are mixed infections with a 1/1 ratio of wild-type and 3D-G64S virus (MOI of 0.1 PFU/cell or 10 PFU/cell for each virus) and a 1/2 ratio (MOI of 5 and 10 PFU/cell, respectively). Products were quantified by Phosphorimager and the percentage 3D-G64S is shown. DOI: 10.1371/journal.ppat.0010011.g003

To test for a defect in RNA accumulation in the 3D-G64S mutant virus, we performed competition assays in HeLa cells with wild-type and G64S mutant virus and monitored the relative proportions of RNA after single-cycle infections. To determine the sensitivity of the competition assay, plasmid DNAs that contained wild-type and 3D-G64S cDNAs were mixed at various ratios and the 3D polymerase coding regions were amplified and end-labeled by PCR. The BstBI-digested products from the wild-type and 3D-G64S genomes (Figure 2A) displayed equivalent intensities when obtained from a 1:1 mixture of wild-type and 3D-G64S mutant cDNAs (Figure 3C). At 1:10 input ratios, however, the bands derived from the minority species were barely visible, and disappeared altogether at 1:100 input ratios. Therefore, this assay is capable of detecting both the wild-type and 3D-G64S-derived products, as long as the input amounts are within 10-fold of each other (Figure 3C). When the relative amounts of RNA accumulation of wild-type and 3D-G64S virus from single and mixed infections of HeLa cell monolayers were monitored, a slight RNA accumulation defect for the 3D-G64S virus was observed. As shown in Figure 3D, a 1:1 input ratio of the two viruses displayed a decreased accumulation of 3D-G64S RNA relative to that of wild-type. Using twice as much 3D-G64S virus in the mixed infection equalized the bands (Figure 3D). Therefore, although no defect was observed in viral growth assays in HeLa cells, 3D-G64S virus displayed a slight RNA accumulation defect during a single cycle of replication.

Viral growth in HeLa cells, however, may not mirror replication capacity in murine cells, and subtle growth defects may be more apparent in primary cells. Therefore, we compared the growth of wild-type and 3D-G64S viruses in mouse embryo fibroblasts (MEF) derived from PVR mice [9]. In single-cycle growth curves in these primary cells, 3D-G64S virus showed slightly reduced accumulation at early time points (Figure 4A), displaying 2- to 4-fold titer reduction compared to wild-type virus at 3, 4, and 5 h post-infection. Despite this slight growth defect in a single-cycle growth curve, wild-type and 3D-G64S viruses displayed similar plaque sizes in the PVR-MEF (Figure 4B). To test whether this slight growth defect would prevent the 3D-G64S virus from propagating during multiple passage in the PVR-MEF, RNA from serially passaged mixtures of wild-type and G64S virus was analyzed. PVR-MEF were inoculated with a 1:1 input ratio of mutant:wild-type virus at high MOI (5–30 PFU/cell). As shown in Figure 4C, the 3D-G64S and wild-type product amounts were comparable over three rounds of serial passage, indicating no significant growth defect exists for the mutant virus in PVR-MEF even during multiple rounds of infection. It should be noted that poliovirus replication is 10- to 100-fold less efficient in MEF compared with HeLa cells (Figures 3A, 4A and 4B); therefore, MEF represent a stringent environment, where growth defects may be more apparent. The fact that 3D-G64S virus matches the replication of wild-type virus in MEF indicates the mutant virus has only a slight growth defect in cultured cells.

**Figure 4:**
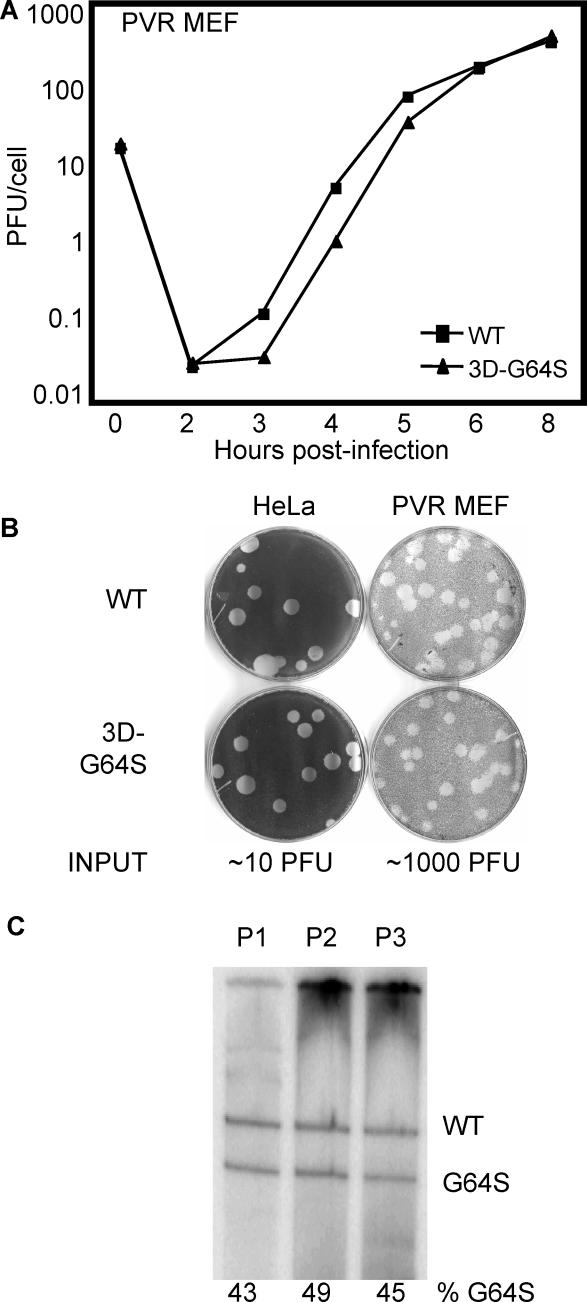
Growth of Wild-Type and 3D-G64S Poliovirus in Primary PVR-MEF (A) Single-cycle growth curve. 2 × 10^5^ PVR-MEF were infected at an MOI of 10 PFU/cell with either wild-type or 3D-G64S viruses at 37 °C. Cell-associated virus was harvested at indicated times and titered by plaque assay. (B) Plaque assays. Wild-type or 3D-G64S poliovirus was plated on HeLa or PVR-MEF monolayers and incubated under an agar overlay for 72 h at 37 °C before staining the cells with crystal violet. The amount of virus plated, according to HeLa titration, is shown underneath as “input.” (C) Serial passage competition assay in PVR-MEF. 3D-G64S and wild-type virus were mixed in a 1:1 ratio and used to infect PVR-MEF at an MOI of 10 PFU/cell at 37 °C. At 6 h post-infection, cell-associated RNA and virus were harvested, and the virus was used to infect fresh cells at high MOI (5–30 PFU/cell). This cycle was repeated a total of three times; BstBI digestion assay products are shown for passages 1, 2, and 3 (P1, P2, P3), and the Phosphorimager-quantified percentage of the G64S product is shown. DOI: 10.1371/journal.ppat.0010011.g004

### Effect of a Defined Selective Pressure on the Fitness of 3D-G64S Mutant Virus: Characterization of the Selective Pressure

The reduced pathogenesis of the 3D-G64S mutant virus could result, as stated previously, from a growth defect or the quasispecies restriction caused by increased polymerase fidelity. In the latter case, the increased mutation rate in the wild-type virus population would presumably aid in viral spread in the diverse environments encountered during spread through the animal. We do not know what these diverse selection pressures might be; therefore, we chose to exert a defined selective pressure on the viral mixtures of wild-type and G64S mutant virus and inquire whether the high-fidelity phenotype of the 3D-G64S virus became a liability. Specifically, a single amino acid change (F28S) in the coding region for poliovirus protein 2C is known to confer temperature sensitivity to the virus in cell culture [16] (J. K. Pfeiffer, unpublished data). We engineered two versions of the F28S amino acid change: one version that contained a single nucleotide substitution [2C-F28S(1m)], which should be able to revert readily during propagation, and a second version that contained three nucleotide substitutions [2C-F28S(3m)], to reduce the probability of reversion to the wild-type codon (Figure 5). These two versions of the F28S mutation were cloned into wild-type and 3D-G64S backgrounds and the resulting viruses were plaque-purified and propagated at 32 °C, the permissive temperature. No differences in the rates of growth of viruses that contained the 2C-F28S(1m) and 2C-F28S(3m) mutations were observed during single-cycle infections in cell culture (J. K. Pfeiffer, unpublished data). As shown in Figure 5, the presence of either the single-nucleotide (1m) or the three-nucleotide (3m) 2C-F28S mutation did not change the ability of the 3D-G64S virus to compete with virus that was wild-type at the 3D locus during serial passage in HeLa cells.

**Figure 5:**
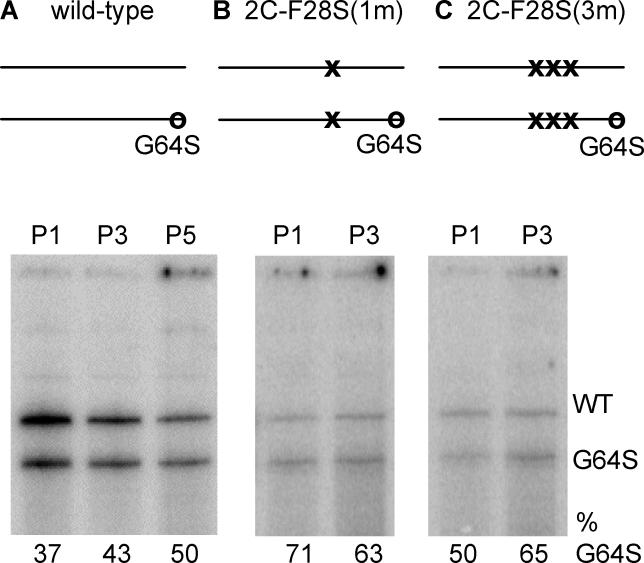
Serial Passage Competition Assay in HeLa Cells for Mixtures of Wild-Type and 3D-G64S Viruses Containing the 2C-F28S Temperature-Sensitive Attenuating Mutation One-to-one mixtures of 3D-G64S to WT virus containing the indicated 2C-F28S mutations were used to infect HeLa cells at an MOI of 0.1 PFU/cell at 32 °C. At 12 h post-infection, cell-associated RNA and virus were harvested, and the virus was titered to determine input for the next passage. This cycle was repeated three to five times, and representative gels of digestion products, with maps indicating the mutations at the 2C locus in the mixtures of viruses that were wild-type and 3D-G64S at the 3D locus, are shown: (A) wild-type, (B) 2C-F28S(1m), (C) 2C-F28S(3m). X denote the numbers of nucleotide substitutions at the 2C-F28S site, while O denotes the presence of the 3D-G64S mutation. DOI: 10.1371/journal.ppat.0010011.g005

To determine the effect of the 2C-F28S(1m) and 2C-F28S(3m) mutations on pathogenesis and growth in mice, and the role of the probability of genetic reversion in these processes, we inoculated PVR mice intramuscularly with the three different mixtures of viruses, designated by their 2C allele, depicted in Figure 5. The three mixtures were generated as follows: *wild-type*, containing a 1:10 mixture of wild-type and 3D-G64S virus; *2C-F28S(1m)*, containing a 1:10 mixture of 2C-F28S(1m) and 2C-F28S(1m)/3D-G64S virus; and *2C-F28S(3m)*, containing a 1:10 mixture of 2C-F28S(3m) and 2C-F28S(3m)/3D-G64S virus (Figure 6A). The ratio of 1:10 (wild-type: 3D-G64S) at the 3D locus was chosen to enhance the detection of 3D-G64S-containing RNAs, should they compete poorly with virus that was wild-type at the 3D polymerase locus. 2-wk-old mice were inoculated by IM injection with the three mixtures of viruses and neuropathological symptoms were monitored as a function of time. Upon injection of 5 × 10^6^ PFU, mice inoculated with virus mixtures that were wild-type at the 2C locus showed symptoms by the third day post-infection, whereas mice inoculated with the 2C-F28S(1m) virus mixture showed a small, but significant, delay in disease onset (Figure 6A). Even more strikingly, the 2C-F28S(3m) virus mixture was found to cause no disease at the input dose of 5 × 10^6^ PFU (Figure 6A). At higher doses, however, the 2C-F28S(3m) virus mixture caused disease (Figure 6B).

**Figure 6:**
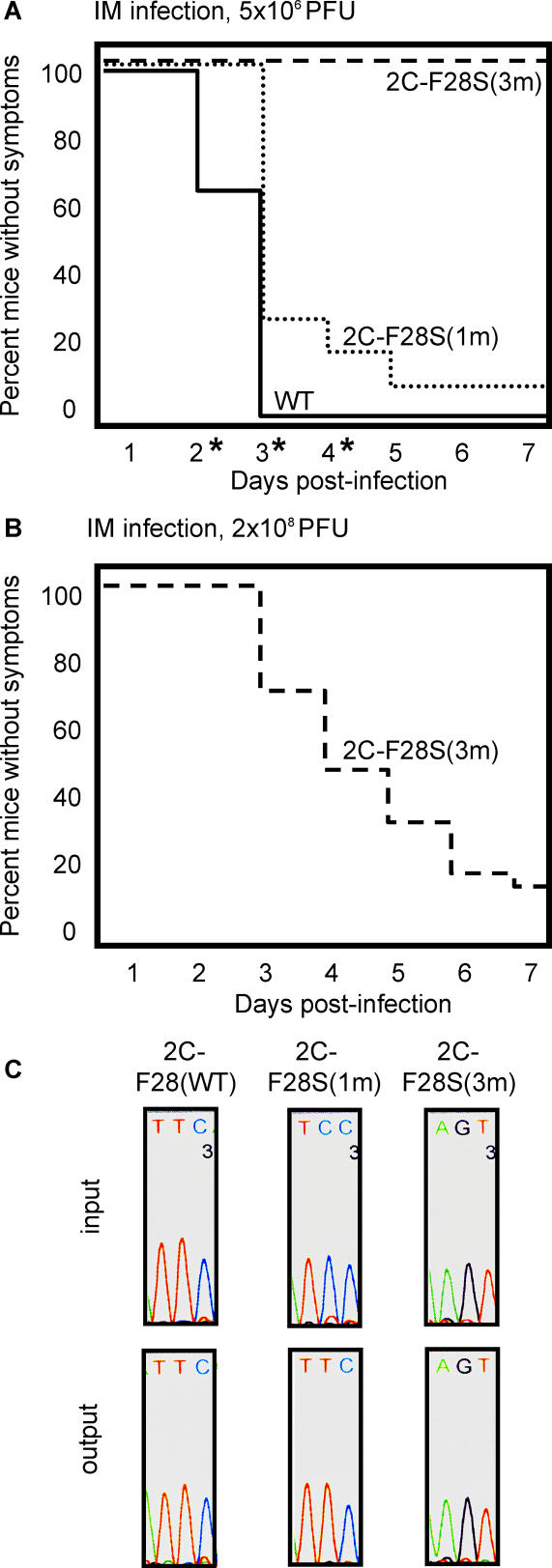
Pathogenesis of Mixtures of Wild-Type and 3D-G64S Viruses Containing the 2C-F28S Temperature-Sensitive Attenuating Mutation PVR mice were injected intramuscularly with 5 × 10^6^ (A) or 2 × 10^8^ (B) PFU of total virus. The viral inocula were 1/10 mixtures of the wild-type/3D-G64S derivatives to allow 3D-G64S viruses a chance to compete. For example, 2C-F28S(1m) was a mixture of 5 × 10^5^ PFU of 2C-F28S(1m) and 4.5 × 10^6^ PFU of 2C-F28S(1m)/3D-G64S, giving a total of 5 × 10^6^ PFU. The numbers of mice in each group, as designated by the identity of the 2C allele, were: wild-type, 26 mice; 2C-F28S(1m), 32 mice; 2C-F28S(3m), 4 and 27 mice in (A) and (B), respectively. Asterisks denote time points where statistically significant differences (*p* < 0.05, determined by two-sample Student's *t-*test, [d 2, 0.00125; d 3, 0.00125; d 4, 0.0112]) were observed between 2C-F28S(1m) and wild-type viruses. (C) Sequence analysis of RT-PCR products from viral RNA pools (“input”) used to inoculate the mice shown in (A) and from muscle tissue after development of disease (“output”). For output samples, samples from at least three mice were sequenced, and a representative example for each is shown. DOI: 10.1371/journal.ppat.0010011.g006

We hypothesized that the 2C-F28S mutation is highly attenuating, as shown by the profoundly reduced pathogenesis of the 2C-F28S(3m) virus mixture. However, because the 2C-F28S(1m) mutations could readily revert to a wild-type sequence, the 2C-F28S(1m) virus mixture caused much more disease than the 2C-F28S(3m) mixture. To test this hypothesis, the viral RNAs present in muscle tissue from diseased mice [wild-type and 2C-F28S(1m) from Figure 6A, and 2C-F28S(3m) from Figure 6B] were amplified and sequenced in the region of the 2C mutations. Portions of the resulting chromatograms, as well as sequences obtained from RT-PCR products of the viral RNAs in the input pools, are shown in Figure 6C. As predicted, the sequences near the site of the mutations at the 2C locus from the wild-type pool, and the 2C-F28S(3m) pool did not change during passage in the mouse. However, virus isolated from mice infected with the 2C-F28S(1m) pool showed a high frequency of reversion to wild-type sequence at the F28S(1m) codon (Figure 6C). Therefore, the increased pathogenesis of the F28S(1m) virus pool, relative to that of the F28S(3m) virus pool, was accompanied by direct reversion of the F28S(1m) mutation, implying that there was strong selective pressure for wild-type sequence at this codon.

### Effect of a Defined Selective Pressure on the Fitness of 3D-G64S Mutant Virus: Growth in Mouse Tissue

To ask whether the presence of the strong selective pressure to revert the 2C-F28C mutation would decrease the competitiveness of 3D-G64S virus, we analyzed the relative ratios of 3D-G64S and wild-type RNA sequence at the 3D locus by analysis of muscle samples from many of the mice shown in Figure 6. For the mixture of viruses that were wild-type at the 2C locus (Figure 7A), the 3D-G64S genomes were more highly represented than genomes that were wild-type at the 3D locus, which was an expected result considering the 10-fold excess of the 3D-G64S virus in the inoculum. However, in the context of the “revertible” attenuating mutation, 2C-F28S(1m), the 3D-G64S products were poorly represented in the resulting population (Figure 7B). However, in the presence of the “non-revertible” 2C-F28S(3m) mutations, the 3D-G64S products were once again well-represented in the population (Figure 7C). These results, quantified in Figure 7D, argue that the high fidelity of the 3D-G64S virus decreases its fitness in the presence of a defined selective pressure, if errors introduced by the wild-type polymerase can bring about escape from that pressure.

**Figure 7:**
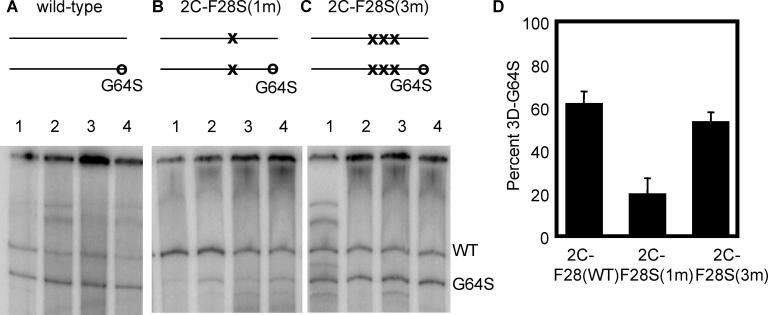
Viral Competition Assay in the Context of the 2C-F28S Temperature-Sensitive Attenuating Mutations Muscle tissue from the experiment shown in Figure 6 was processed and the BstBI digestion assay was performed. Representative gels of digestion products, with maps indicating the mutations at the 2C locus are shown: (A) wild-type, (B) 2C-F28S(1m), (C) 2C-F28S(3m). (D) Quantitation of the gels in A–C. The percent 3D-G64S is shown, and represents an average from 5–10 mice for each condition. DOI: 10.1371/journal.ppat.0010011.g007

## Discussion

It has been theorized that RNA viruses need high error rates to survive in complex environments [1]. With the isolation of a high-fidelity poliovirus, we were in a unique position to address this hypothesis.

In tissue culture, we found a slight RNA accumulation defect of 3D-G64S poliovirus in HeLa cells (Figure 3), which may result from the decreased rate of nucleotide incorporation observed in reactions with purified 3D-G64S polymerase [8]. In murine embryo fibroblasts derived from PVR-expressing mice, a slight growth defect was also observed in single-cycle infections (Figure 4A). Nevertheless, in both HeLa cells and PVR-MEF, the 3D-G64S could compete with wild-type virus over several rounds of serial passage (Figures 4 and 5). Our results differ from those of Arnold et al, where population sequencing suggested the 3D-G64S virus could not compete with wild-type virus even during one round of competition passage [8]. We do not know the basis for this discrepancy, but it could be due to differences in the virus clones used, the method of detection, or the cell lines.

In mouse infections, the 3D-G64S virus was less pathogenic than wild-type virus, as measured by the time of symptom onset and the number of animals acquiring disease (Figure 1). When mice were inoculated with a mixture of the wild-type and 3D-G64S viruses, both were able to replicate in muscle and both were capable of spreading to the brain, although 3D-G64S virus spread to the brain less often than wild-type virus. Interestingly, we found that generally, only one of the two input viruses was present in the brain of infected animals. This so-called poliovirus “bottleneck effect” has been observed before, both in mice and in humans [14,15]. Other pathogens also encounter bottlenecks during spread within a host. The plant RNA virus Cucumber mosaic virus undergoes striking bottlenecks during spread within an infected plant [17], and *Salmonella* strains and *Pneumocystis carnii* experience bottlenecks during spread in rodents [18–20]. We will describe the nature of the poliovirus bottleneck effect in more detail in the future.

The virulence and growth defects of 3D-G64S poliovirus in mice could be due to its enhanced fidelity or to the slight growth defect documented here. To determine whether enhanced fidelity could play a role in the fitness of poliovirus, we competed 3D-G64S virus against virus that was wild-type at the 3D locus in the context of a defined selective pressure, a revertible attenuating point mutation in the viral 2C coding region. Indeed, the 3D-G64S virus was unable to compete with virus that was wild-type at the 3D locus in the presence of a revertible version of the 2C mutation. This did not result from a growth defect of the 3D-G64S virus, because, in the absence of the 2C mutation, or in the presence of a non-revertible version of the 2C mutation, the 3D-G64S-containing virus could compete well with virus that was wild-type at the 3D polymerase locus (Figure 7).

The results presented here argue that a high-fidelity poliovirus variant that contains the 3D-G64S mutation in the coding region for its RNA-dependent RNA polymerase is less able to revert attenuating point mutations. Therefore, in the context of known, harmful, mutations, a virus that is wild-type at the 3D polymerase locus is more fit than the higher-fidelity 3D-G64S virus. Most of the mutations made during viral growth are likely to be deleterious, and it is possible that reversion of attenuating mutations is important for survival of RNA viruses. However, why would it not be advantageous for a virus to encode a higher-fidelity polymerase and simply not to make so many errors in the first place?

Genetic change can be very rapid in the presence of a low-fidelity polymerase (Figure 6C). In a complex environment such as within a mouse, the ability to generate a quasispecies may allow virus populations to respond to the different environments they encounter during spread between tissues and in reaction to changing pressure of the host immune response. In monkeys infected with a vaccine strain of poliovirus, distinct mutations were observed to occur within 2 d post-infection, only to revert to the inoculated sequence later in the course of disease [21]. Furthermore, HIV is known to change its cell tropism from macrophage-tropic to T cell-tropic, during spread in many infected individuals [22–23]. It would be interesting to determine whether the pathogenesis of HIV is reduced by increasing the fidelity of its replicative machinery. We have shown that, in the presence of a defined selective pressure, the increased fidelity of the 3D-G64S mutant virus decreases its fitness. Given the slight growth defect of the 3D-G64S mutant virus, we further suggest that, the decreased virulence of high-fidelity 3D-G64S mutant poliovirus in the mouse is due, at least in part, to its relative inability to generate variants suitable for the variety of selection pressures faced during the normal course of infection of a complex organism.

## Materials and Methods

### Cells, mice, and viruses.

HeLa cells were grown in Dulbecco's modified Eagle's medium, supplemented with 10% calf serum [24]. PVR mice expressing the human poliovirus receptor (CD155) from a β-actin promoter were obtained from R. Andino (University of California at San Francisco) [9]. Primary MEF were harvested from PVR mice. Day 14–18 embryos were harvested, washed in phosphate-buffered saline, the heads and viscera were removed, and the body was minced with a sterile razor blade. The slurry was trypsinized and homogenized with a pipet for 10 min and then plated in Dulbecco's modified Eagle's medium, supplemented with 10% fetal bovine serum. Experiments with MEF were performed between passages two and five (see below). Mahoney serotype 1 poliovirus was grown from a single plaque generated by transfection of the viral cDNA clone [25]. Individual plaques were grown into high-titer virus stocks by infecting monolayers of HeLa cells at 37 °C. Plasmids that encoded cDNAs for temperature-sensitive mutant viruses were transfected at 32 °C, and small plaques were picked 4 d later. In all cases, care was taken to ensure that the specific infectivities were comparable to wild-type plasmid cDNAs transfected at the same time. Multiple individual plaques were chosen from the transfections and propagated for three cycles of infection at 32 °C to generate high-titer stocks. The phenotypes of these stocks were then compared to those of the original plaque isolates to ensure that no additional mutations were acquired during propagation.

### Plasmid construction.

The 3D-G64S mutation in the viral 3D-coding region was introduced by site-specific mutagenesis, which added a BstBI restriction enzyme site (sense primer: 5′ATTTTCTCCAAGTACGTT*TCG*AACAAAATTACTGAAGTG 3′). This BstBI-marked 3D-G64S derivative was engineered to contain three nucleotide substitutions (GGT to TCG) to minimize reversion at this site. A mutation in the 2C coding region, F28S, which confers temperature sensitivity [16], was introduced into both the 3D-G64S and wild-type poliovirus clones in two forms: one with nucleotide substitution [TTC to TCC, termed *2C-F28S(1m)*] and the other with three nucleotide substitutions [TTC to AGT, termed *2C-F28S(3m)*]. In each case, the entire PCR-generated region was confirmed by sequencing.

### Tissue culture infections.

For single-cycle viral growth curves, 2 × 10^6^ HeLa cells or 2 × 10^5^ PVR-MEF were infected at 37 °C at an MOI of 10 PFU/cell, harvested at various times post-infection, and lysed by repeated freeze-thawing. Cell-associated virus was quantified by plaque assay on HeLa cell monolayers [7]. Each growth curve was performed at least twice and results were similar in all replicates. For competition experiments, as shown in Figure 3D, 2 × 10^6^ HeLa cells were infected at an MOI of 0.1 or 10 PFU/cell with wild-type virus, 3D-G64S virus, or both viruses, at varying ratios for 5 h at 37 °C. Cells were harvested and RNA was extracted using TRIZOL (Invitrogen, Carlsbad, California, United States) according to the manufacturer's instructions. For serial passage competition experiments shown in Figure 5, 2 × 10^6^ HeLa cells were infected at 32 °C with a 1:1 mixture of wild-type and 3D-G64S viruses at low MOI (0.1 PFU/cell). At 12 h post-infection, cell-associated virus and RNA were harvested (as above), and the cycle was repeated three to five times, titering the virus each time to determine input for the next cycle. Serial passage experiments in PVR-MEF (Figure 4C) were performed as above, except that 2 × 10^6^ MEF were infected at a high MOI (5–30 PFU/cell) at 37 °C, and cell-associated virus and RNA were harvested at 6 h post-infection.

### Mouse infections and tissue processing.

All animal work was performed according to protocols approved by the Stanford University Administrative Panel on Laboratory Animal Care. For IM inoculations, 50 μl of virus was injected into the thigh muscle of the right hind leg of mice that were 2–10 wk old, as indicated, using a 28–gauge needle. Before IC inoculations, 2-wk old animals were anesthetized by intraperitoneal injection with 11 μl of a solution containing 2.5% avertin/g animal weight. Once animals were unconscious, 20 μl of virus stock was injected into the cerebrum through the right temple. Mice were observed for symptoms twice per day, and were euthanized at the first sign of disease, weakness in the inoculated limb, or, for IC-inoculated mice, ruffled fur and general lethargy. Tissues, including the inoculated leg muscle, brain, or both, were harvested and frozen at −80 °C until studied. Tissues were ground into a fine powder under liquid nitrogen using a mortar and pestle. RNA was extracted from approximately 300 mg of total tissue using TRIZOL (Invitrogen) according to the supplied protocol.

### Reverse transcription-PCR digestion assay.

RNA was reverse transcribed using 5–10 μg of RNA per cDNA synthesis reaction using Superscript II reverse transcriptase (Invitrogen) and reverse primer 5′ATAGGTTCCCAGAA GCCATTCTC3′ for the 3D polymerase-coding region or 5′CCATGTCGAACGCAGAGCGCCTGG3′ for the 2C-coding region. Twenty percent of the reverse transcription reaction was PCR amplified using 5′CCGCATTTGGAAGTGGATCTACTCAGCAG3′ and 5′CCATGTCGAACGCAGAGCGCCTGG3′ primers for the 2C-coding region and primers 5′GCTTCACCTGGTGAAAGCATTGTGATCG3′ and 5′ATAGGTTCCCAGAA GCCATTCTC3′ primers for the 3D-coding region. The 3D reverse primer was end-labeled using T4 polynucleotide kinase (New England Biolabs, Beverly, Massachusetts, United States), and 125 μCi [γ-^32^P] ATP (Perkin-Elmer, Boston, Massachusetts, United States), and was used at a 1:5 ratio with the unlabeled 3D reverse primer in PCR reactions. The PCR reactions contained 1 unit Taq (Qiagen, Valencia, California, United States) and were done according to the supplier protocol, using a buffer that contained 50 μM potassium glutamate, 20 μM potassium HEPES (pH 8.4), 3 μM MgCl_2_ and 0.1 mg/ml BSA. The PCR products were digested with BstBI (New England Biolabs) at 65 °C for 3–16 h, and products were ethanol-precipitated in the presence of glycogen (Invitrogen). Pellets were resuspended in denaturing gel loading dye (80% formamide, 10 mM EDTA, 0.025% bromophenol blue and xylene cyanol, 0.1% SDS), heated to 80 °C for 2 min, and loaded on gels that contained 7% polyacrylamide and 8M urea. Gels were dried under vacuum on DE81 paper (Whatman, Florham Park, New Jersey, United States) and exposed to PhosphorImager screens.

### Sequence analysis.

The cDNAs products from viral RNAs purified using the QiaAmp kit (Qiagen) or from cDNA-encoding plasmids were PCR-amplified for sequence analysis at the F28S locus. The PCR reactions were performed as above, except that no radiolabel was added. Products were run on 1% agarose gels and products were excised, purified using the Qiaex II kit (Qiagen), and sequenced commercially (Sequetech, Mountain View, California, United States).

### Accession Number

The Genbank (http://www.ncbi.nlm.nih.gov/entrez) accession number for serotype 1 poliovirus is NC002058.

## Abbreviations

bp: basepair
IC: intracerebral
IM: intramuscular
MEF: mouse embryo fibroblast
MOI: multiplicity of infection
PFU: plaque-forming unit
PVR: poliovirus receptor
